# “Antedisciplinary” Science

**DOI:** 10.1371/journal.pcbi.0010006

**Published:** 2005-06-24

**Authors:** Sean R Eddy

“The scale and complexity of today's biomedical research problems demand that scientists move beyond the confines of their individual disciplines and explore new organizational models for team science. Advances in molecular imaging, for example, require collaborations among diverse groups—radiologists, cell biologists, physicists, and computer programmers.” —National Institutes of Health Roadmap Initiative [1]

Reading this made me a little depressed. For starters, the phrase “organizational models for team science” makes me imagine a factory floor of scientists toiling away on their next 100-author paper under the watchful gaze of their National Institutes of Health program officers, like some scene from Terry Gilliam's movie *Brazil*. It's also depressing to read that the National Institutes of Health thinks that science has become too hard for individual humans to cope with, and that it will take the hive mind of an interdisciplinary “research team of the future” to make progress. But what's most depressing comes from purely selfish reasons: if groundbreaking science really requires assembling teams of people with proper credentials from different disciplines, then I have made some very bad career moves.

I've been a computational biologist for about 15 years now. We're still not quite sure what “computational biology” means, but we seem to agree that it's an interdisciplinary field, requiring skills in computer science, molecular biology, statistics, mathematics, and more. I'm not qualified in any of these fields. I'm certainly not a card-carrying software developer, computer scientist, or mathematician, though I spend most of my time writing software, developing algorithms, and deriving equations. I do have formal training in molecular biology, but that was 15 years ago, and I'm sure my union card has expired. For one thing, they all seem to be using these clever, expensive kits now in my wet lab, whereas I made most of my own buffers (after walking to the lab six miles in the snow, barefoot).

If I thought I was the only person who abandoned disciplinary training to take up a new area of science, after reading about the “research teams of the future,” I might slink away and find something else to do before the future arrives. But I don't think I'm alone. I was recently at a meeting where people started discussing these interdisciplinary “research teams of the future,” and Howard Berg, who had just given a wonderful chalk talk about bacterial chemotaxis, was sitting behind me. I heard him mutter that he wondered how a misfit like him was going to fit into this new world order. Well, he's doomed. He's successfully applied physical, mathematical, and biological approaches to an important problem without enlisting an interdisciplinary team of properly qualified physicists, mathematicians, and biologists. As he recently wrote, perhaps he'll have to start collaborating with himself [2].

I wonder if it's the success of the Human Genome Project that led us to this. The scale of the genome project required “big science” and large teams. The genome project also fueled the explosive growth of the highly successful field of computational biology. Did the ideas of interdisciplinary science and large teams become inappropriately intertwined? Certainly, achieving the goals of the Human Genome Project required engineers, physicists, and computer scientists. It would be silly to argue against large interdisciplinary teams where a mammoth technical goal can be clearly defined. But when I think of new fields in science that have been opened, I don't think of interdisciplinary *teams* combining existing skills to solve a defined problem—I think of single interdisciplinary *people* inventing new ways to look at the world.

Focusing on interdisciplinary teams instead of interdisciplinary people reinforces standard disciplinary boundaries rather than breaking them down. An interdisciplinary team is a committee in which members identify themselves as an expert in something else besides the actual scientific problem at hand, and abdicate responsibility for the majority of the work because it's not their field. Expecting a team of disciplinary scientists to develop a new field is like sending a team of monolingual diplomats to the United Nations.

Progress is driven by new scientific questions, which demand new ways of thinking. You want to go where a question takes you, not where your training left you. We may not have a single clarion call to arms like Schrödinger's *What is Life?* driving physicists into biology right now, as in the beginnings of molecular biology. But we do have powerful new technologies to harness (computational biology), newly revitalized approaches to old problems (systems biology), and new areas altogether (synthetic biology). New disciplines eventually self-organize around new problems and approaches, creating a new shared culture. This shared culture coalesces into the next essential training regimen for the next generation of scientists, and with luck, some of these people will overcome their training to open up more new fields of inquiry. Interdisciplinary science is just the embryonic stage of a new discipline. To value interdisciplinary science for its own sake is to value history over progress—that is, to value people's past training more than their current work.

Don't get me wrong. Certainly experience does affect how problems are approached, and it's synergistic to bring together people with different ideas. It's just a question of emphasis. In a marriage, previous experience affects what is brought to the partnership; but dwelling too much on prior experience causes the commitment to the current project to be called into question. Show me someone working on modeling the yeast cell cycle who still calls himself a physicist, and I'll show you someone with commitment issues.

Consider, for instance, the rise of molecular biology as a discipline. We think of Watson and Crick as molecular biologists, not as an ornithologist and a physicist. The first molecular biologists were a motley crew of misfits and revolutionaries with no particularly relevant training, many of them ex-physicists. These physicists didn't waste much time identifying themselves as physicists any more. They viewed themselves as a new kind of biologist. They burned their bridges. Max Delbrück dropped physics and started studying phage replication because it seemed like the fastest, best way to crack the molecular basis of heredity. It's hard to imagine molecular biology making such dramatic progress if it had involved forming interdisciplinary teams of physicists and biologists. The molecular biologists were viewed as naive infidels. Biochemist Erwin Chargaff sniffed that “molecular biology is the practice of biochemistry without a license” [3].

Molecular biologists even worried about what to call themselves, like we argue over whether we're computational biologists or bioinformaticians. Any revolution needs to find the right slogan to unify under. Francis Crick explained, “I myself was forced to call myself a molecular biologist because when inquiring clergymen asked me what I did, I got tired of explaining that I was a mixture of crystallographer, biophysicist, biochemist, and geneticist, an explanation which in any case they found too hard to grasp” [4].

To encourage the rise of new disciplines as successful as molecular biology, we need to encourage individuals to leave old disciplines behind and forge new fields. New science needs to be judged on its merits, not by the disciplinary credentials of the people doing it—particularly in fast-moving interdisciplinary areas where any formal training may be outdated anyway. If your grant proposal includes statistical analysis, your reviewers shouldn't be acting as enforcers requiring you to have a card-carrying statistician as a collaborator. Maybe in your narrow area, you know how to do the relevant statistics as well as any formally trained statistician. A proposal invoking high-performance computing should not get held up until you enlist collaborating computer scientists, who may not even be interested in your problem. Maybe you know how to use a supercomputer well enough to do what you propose.

Perhaps the whole idea of interdisciplinary science is the wrong way to look at what we want to encourage. What we really mean is “antedisciplinary” science—the science that precedes the organization of new disciplines, the Wild West frontier stage that comes before the law arrives. It's apropos that antedisciplinary sounds like “anti-disciplinary.” People who gravitate to the unexplored frontiers tend to be self-selected as people who don't like disciplines—or discipline, for that matter.

One can't deny that science is getting more complex, because the sheer amount of knowledge is growing. But the history of science is full of ideas that seemed radical, unfathomable, and interdisciplinary at the time, but that now we teach to undergraduates. Every generation, we somehow compress our knowledge just enough to leave room in our brains for one more generation of progress. This is not going to stop. It may take big interdisciplinary teams to achieve certain technical goals as they come tantalizingly within view, but someone also needs to synthesize new knowledge and make it useful to individual human minds, so the next generation will have a taller set of giants' shoulders to stand on. Computer science mythologizes the big teams and great computing engines of Bletchley Park cracking the Enigma code as much as we mythologize the Human Genome Project, but computer science rests more on the lasting visions of unique intellectual adventurers like Alan Turing and John von Neumann. Looking around my desk at the work I'm trying to build on, I do see the human genome paper, but even more, I see the work of individual pioneers who left old disciplines and defined new ones—writing with the coherence, clarity, and glorious idiosyncrasy that can only come from a single mind.
